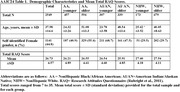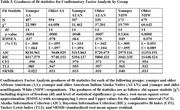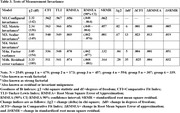# How well does the RAQ measure up? Assessing attitudes toward research in a diverse community sample

**DOI:** 10.1002/alz.088524

**Published:** 2025-01-09

**Authors:** Diane Carol Gooding, C. Malik Boykin, Shenikqua Bouges, Carol A. Van Hulle, Jordan P. Lewis, Megan L. Zuelsdorff, Fabu P. Carter, Carey E. Gleason

**Affiliations:** ^1^ Department of Psychiatry, University of Wisconsin‐Madison, School of Medicine and Public Health, Madison, WI USA; ^2^ Division of Geriatrics and Gerontology, Department of Medicine, University of Wisconsin‐Madison, School of Medicine and Public Health, Madison, WI USA; ^3^ Department of Psychology, University of Wisconsin‐Madison, College of Letters & Science, Madison, WI USA; ^4^ Brown University, Providence, RI USA; ^5^ Dept of Cognitive, Linguistic, and Psychological Sciences, Brown University, Providence, RI USA; ^6^ Center for Study of Race and Ethnicity in America, Brown University, Providence, RI USA; ^7^ Data Science Institute, Brown University, Providence, RI USA; ^8^ Wisconsin Alzheimer’s Disease Research Center, University of Wisconsin School of Medicine and Public Health, Madison, WI USA; ^9^ Madison VA GRECC, William S. Middleton Memorial Hospital, Madison, WI USA; ^10^ Division of Geriatrics and Gerontology, University of Wisconsin‐Madison, School of Medicine and Public Health, Madison, WI USA; ^11^ Wisconsin Alzheimer’s Institute, University of Wisconsin‐Madison, School of Medicine and Public Health, Madison, WI USA; ^12^ Memory Keepers Medical Discovery Team, University of Minnesota Medical School, Duluth campus, Duluth, MN USA; ^13^ Department of Family Medicine & Biobehavioral Health, University of Minnesota Medical School, Duluth, MN USA; ^14^ School of Nursing, University of Wisconsin‐ Madison, Madison, WI USA; ^15^ Division of Geriatrics, Department of Medicine, University of Wisconsin School of Medicine and Public Health, Madison, WI USA; ^16^ Wisconsin Alzheimer's Disease Research Center, University of Wisconsin School of Medicine and Public Health, Madison, WI USA; ^17^ Wisconsin Alzheimer’s Institute, University of Wisconsin School of Medicine and Public Health, Madison, WI USA

## Abstract

**Background:**

Individuals’ attitudes toward research predict recruitment, engagement, and retention. The Research Attitudes Questionnaire (RAQ), developed to predict individuals’ willingness to participate, is often used in AD research. It can be used to identify strategies to mitigate individuals’ reluctance to engage in research. To date, there are mixed findings regarding diverse groups’ willingness to engage in AD research. Between‐group comparisons are only meaningful and valid when the measures being used reflect true group differences in the construct i.e., measurement invariance (MI). One of the prerequisites for meaningful comparisons across diverse groups is to have measurement invariance. The study goal was to examine the suitability of the RAQ across age and ethno‐racialized identities.

**Methods:**

We explored the MI of the 7‐item RAQ using Confirmatory Factor Analysis (CFA) via Mplus in a community‐derived sample of 457 younger and 594 older African American, 307 younger and 339 older American Indian/Alaska Native, and 173 younger and 679 older Non‐Hispanic White male and female individuals. CFA evaluated the one‐factor model across the groups. Subsequently, loadings, means, and residuals were consecutively constrained to equality. We examined whether the increased restrictions produced significant changes in the data‐model fit to determine whether conditions were met to demonstrate varying levels of measurement invariance.

**Results:**

Table 1 summarizes sample demographics and provides mean total RAQ scores. The RAQ showed good internal consistency across age and ethno‐racialized groups (Cronbach’s a ranging from 0.78 to .85). A one‐factor model showed acceptable fit across the groups (Table 2). The results of the cross‐sample invariance tests are provided in Table 3. We found evidence of configural invariance; comparisons of successive models supported metric invariance, and scalar invariance. Strict invariance was not supported.

**Conclusions:**

These data suggest that across the 6 groups, who differed in terms of age and ethno‐racial identity, the strengths of associations between the specific scale items and the latent construct being assessed by the RAQ are the same. However, the groups may differ in the extent to which they are characterized by the latent variable. This finding supports the suitability of the RAQ for cross‐cultural comparisons of willingness to engage in research.